# Supplementary material for: Causal Relationship between Aging and Anorexia Nervosa: A White-Matter-Microstructure-Mediated Mendelian Randomization Analysis
Source: Biomedicines. 2024 Aug 16;12(8):1874. doi: 10.3390/biomedicines12081874 (PMC11351342; doi:10.3390/biomedicines12081874)
Supplement: Supplementary file 1 [file biomedicines-12-01874-s001.zip › Supplementary Figure/Figure S4/Singe SNP plot of MR analysis for TL to ubm-b-1454.pdf]

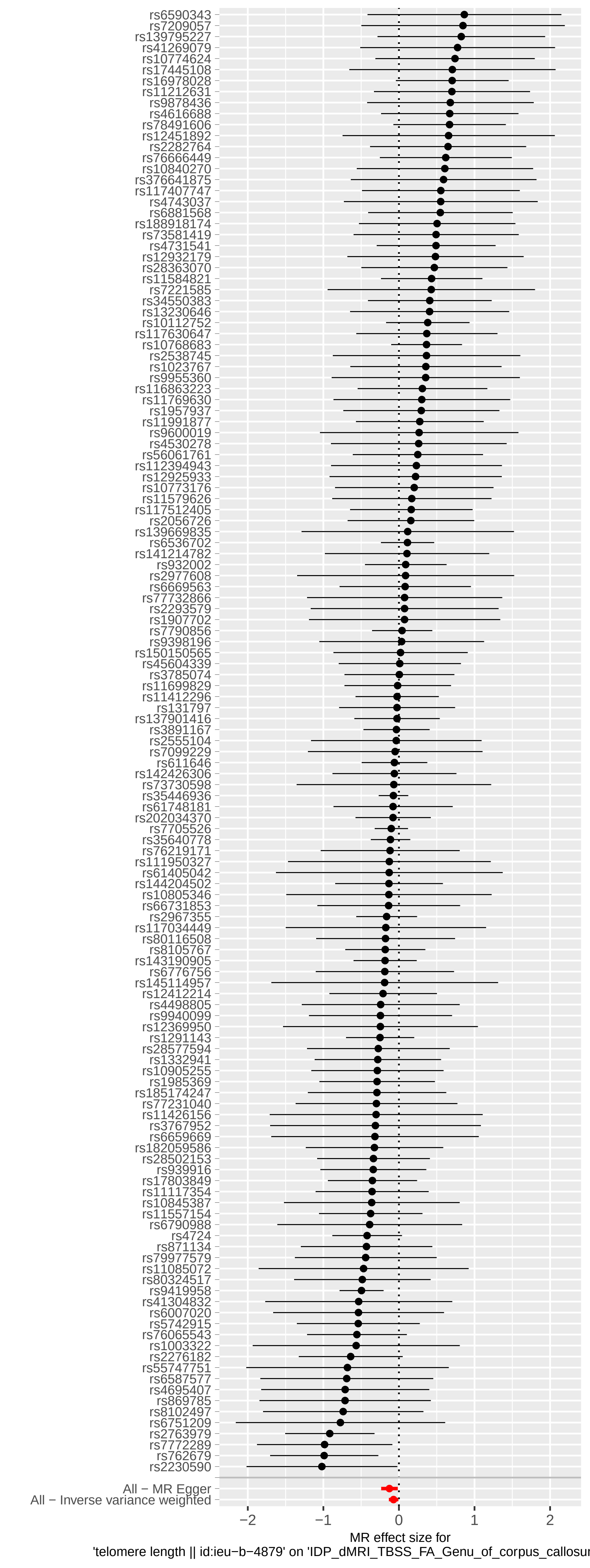
rs6590343
rs7209057
rs139795227
rs41269079
rs10774624
rs17445108
rs16978028
rs11212631
rs9878436
rs4616688
rs78491606
rs12451892
rs2282764
rs76666449
rs10840270
rs376641875
rs117407747
rs4743037
rs6881568
rs188918174
rs73581419
rs4731541
rs12932179
rs28363070
rs11584821
rs7221585
rs34550383
rs13230646
rs10112752
rs117630647
rs10768683
rs2538745
rs1023767
rs9955360
rs116863223
rs11769630
rs1957937
rs11991877
rs9600019
rs4530278
rs56061761
rs112394943
rs12925933
rs10773176
rs11579626
rs117512405
rs2056726
rs139669835
rs6536702
rs141214782
rs932002
rs2977608
rs6669563
rs77732866
rs2293579
rs1907702
rs7790856
rs9398196
rs150150565
rs45604339
rs3785074
rs11699829
rs11412296
rs131797
rs137901416
rs3891167
rs2555104
rs7099229
rs611646
rs142426306
rs73730598
rs35446936
rs61748181
rs202034370
rs7705526
rs35640778
rs76219171
rs111950327
rs61405042
rs144204502
rs10805346
rs66731853
rs2967355
rs117034449
rs80116508
rs8105767
rs143190905
rs6776756
rs145114957
rs12412214
rs4498805
rs9940099
rs12369950
rs1291143
rs28577594
rs1332941
rs10905255
rs1985369
rs185174247
rs77231040
rs11426156
rs3767952
rs6659669
rs182059586
rs28502153
rs939916
rs17803849
rs11117354
rs10845387
rs11557154
rs6790988
rs4724
rs871134
rs79977579
rs11085072
rs80324517
rs9419958
rs41304832
rs6007020
rs5742915
rs76065543
rs1003322
rs2276182
rs55747751
rs6587577
rs4695407
rs869785
rs8102497
rs6751209
rs2763979
rs7772289
rs762679
rs2230590
All − MR Egger
All − Inverse variance weighted
−2
−1
0
1
2
MR effect size for
'telomere length || id:ieu−b−4879' on 'IDP_dMRI_TBSS_FA_Genu_of_corpus_callosu
